# Administration of a second generation perfluorochemical in combination with hyperbaric oxygenation does not provide additional benefit in a model of permanent middle cerebral artery occlusion in rats

**DOI:** 10.1186/2193-1801-3-32

**Published:** 2014-01-17

**Authors:** Ulf C Schneider, Tobias Karutz, Lothar Schilling, Johannes Woitzik

**Affiliations:** Department of Neurosurgery, Charité - Universitätsmedizin Berlin, Berlin, Germany; Center for Stroke Research Berlin, Berlin, Germany; Department of Neurosurgery, University Hospital Mannheim, Mannheim, Germany

**Keywords:** Experimental stroke, Focal cerebral ischemia, Laser Doppler flowmetry, Perfluorochemical, Hyperbaric oxygenation

## Abstract

**Objective:**

Both, second generation perfluorochemicals (Oxycyte®) and hyperbaric oxygen (HBO) have been shown to reduce necrotic tissue volume if administered early after experimental cerebral ischemia. With the idea of exponentiation of oxygen delivery to ischemic tissue, this study was conducted to investigate the combined effect of both treatment modalities on the extent of ischemic brain damage.

**Methods:**

Permanent focal cerebral ischemia was induced in rats by middle cerebral artery occlusion (MCAO). Animals were assigned randomly to one of the following treatment groups: Control (0.9% NaCl, 1 ml/100 g i.v.), Oxycyte® (1 ml/100 g i.v.), HBO (1 bar hyperbaric oxygenation for 1 h) and HBO + Oxycyte® (1 ml/100 g i.v. combined with 1 bar hyperbaric oxygenation for 1 h). Injection of NaCl or Oxycyte® was performed following MCAO. After injection, breathing was changed to 100% oxygen in Oxycyte®-, HBO- and HBO + Oxycyte®-groups. After eight hours the necrotic volume was calculated from serial coronal sections stained with silver-nitrate and corrected for the extent of swelling.

**Results:**

Hemodynamic and metabolic parameters were not affected by infusion of Oxycyte®. Total necrosis volume was significantly reduced in HBO-treated animals (223 ± 70 mm^3^), when compared to control animals (335 ± 36 mm^3^). In animals after Oxycyte®-treatment alone (299 ± 33 mm^3^) or combined HBO + Oxycyte®-treatment (364 ± 50 mm^3^) did not show a significantly smaller necrosis volume compared to control animals (necrosis volumes are given as mean ± SD).

**Discussion:**

These results suggest that combination of hyperbaric oxygenation and Oxycyte® administered immediately after onset of vascular occlusion does not provide an additional neuroprotective effect in the early phase of brain ischemia.

## Introduction

Perfluorochemicals (PFCs) are emulsified artificial oxygen carriers that dissolve oxygen physically in a linear function, dependant on the partial oxygen pressure. Due to technological advances in the emulsification process a total oxygen binding capacity comparable to that of hemoglobin can be reached in newly designed so-called second generation PFCs (approximately 17–20 volume % at 1 bar oxygen pressure). If administered intravenously they have been shown to improve the capability of oxygen transport in the blood remarkably (Suyama et al., 1981; Sutherland et al., 1984; Kolluri et al., 1986a, 1986b). PFCs have been used in experimental settings of focal cerebral ischemia to improve oxygen delivery to the endangered tissue with the effect of a significant reduction of infarct volume (Peerless et al., 1981; Woitzik et al., 2005).

Another possible treatment for ischemic stroke that has been used in various experimental and clinical settings is hyperbaric oxygen (HBO) therapy. Although its neuroprotective mechanisms are still poorly understood and its effectiveness is still under discussion, proponents suggest that early human research and more recent animal data demonstrate its effectiveness in the early treatment of ischemic stroke (Sunami et al., 2000). Although other molecular mechanisms comprising hypoxia inducible factor 1α (HIF 1α) and its target genes, might play pivotal roles, the most straightforward idea of the neuroprotective effect of HBO might be the improved oxygenation of the tissue at risk (Ostrowski et al., 2005; Calvert et al., 2006; Gu et al., 2008; Matchett et al., 2009).

Due to the linear dissolubility-curve of oxygen in PFC-solutions dependant on the partial oxygen pressure, the oxygen concentration in PFC-enhanced blood can be potentiated by applying HBO (Mitsuno et al., 1984, 1988).

To test the hypothesis of oxygen delivery-induced neuroprotection we combined these two treatment strategies with the idea to further increase oxygen delivery to the endangered tissue. Since hypoxia is presumed to occur in the early phase of ischemia predominantly, we used a short time-course of ischemia, i.e. an eight hours permanent MCA occlusion protocol in rats.

## Materials and methods

The experimental protocol was approved by the federal animal Ethics Committee (Regierungspräsidium Karlsruhe) and performed according to the institutional guidelines and state laws.

### Operative procedure

Male Sprague–Dawley rats (body weight (bw), 270–350 g) were anesthetized with 2% isoflurane delivered through a face mask. Body temperature was kept constant at 37°C using a rectal temperature-controlled heating pad. All animals received a subcutaneous injection of 5 μg/100 g bw atropine (Fresenius, Bad Homburg, Germany) and 3 μg/100 g bw buprenorphine (Temgesic™, Essex Pharma, Munich, Germany) to reduce mucus production and postoperative pain, respectively.

As described before, animals were placed in a stereotactic frame (Kopf Instruments, Tujunga, USA) and two burr holes were drilled in the skull over the right hemisphere leaving the dura intact. Positions of the burr holes were 2 mm posterior and 4 mm lateral, and 4 mm posterior and 4 mm lateral to the bregma, respectively. In each burr hole, a laser Doppler flowmetry (LDF) fiber (diameter, 0.4 mm) was positioned and fixed with cyanacrylate glue and dental cement, which also led to a sealing of the incision. Cerebral blood flow (CBF) was continuously monitored (DRT4, Moor Instruments, Devon, England) and recorded using a LabView (National Instruments, Munich, Germany) based multimodal monitoring system developed in our laboratory. Initial sampling rate was 150 Hz, and data were sampled down to 1 Hz for analysis (Woitzik et al., 2005). Additionally, arterial blood pressure was continuously monitored via an arterial line in the femoral artery.

Animals were turned to a supine position for induction of a focal cerebral ischemia by intraluminal occlusion of the MCA as described previously (Takano et al., 1997; Woitzik and Schilling, 2002). Briefly, the right common carotid artery (CCA) was exposed through a midline incision and carefully dissected from the surrounding tissue and vagal nerve using microsurgery techniques. The external carotid artery, the lingual and the maxillary artery were ligated and divided and the internal carotid artery (ICA) was exposed. A silk suture was tied loosely around the ICA and a silicon coated nylon suture (4–0) was introduced into the CCA via a small incision. For coating of the suture a silicon elastomer (Provil, Bayer Dental, Leverkusen, Germany) was applied over a distance of 5 mm to give the tip a diameter of 430 to 460 μm. The diameter was carefully controlled using a stereomicroscope (GZ6, Leica, Bensheim, Germany) equipped with a scale in an eyepiece (10 μm scaling). The suture was advanced with special care not to enter the pterygopalatine artery until an abrupt drop of the LDF signal indicated MCA occlusion. The suture was fixed to avoid shifting (Woitzik et al., 2011) and the neck incision was closed. After removing the LDF fibers from the skull anesthesia was discontinued and the animals were allowed to recover.

### Treatment groups

Animals were randomly assigned to one of four different treatment regimes starting directly after MCA occlusion (1: control-group, 2: HBO-group, 3: Oxycyte®-group and 4: HBO + Oxycyte®-group). A total of 20 animals (5 per group) was used for this study. Sample size calculations were performed prior to the commencement of the study and were based on data from a previous study (Woitzik et al., 2005). The study was powered to detect a previously reported 12% difference in infarct volume between control and Oxycyte® treatment groups (Woitzik et al., 2005). The control-group and the HBO-group received an intravenous (i.v.) application of isotonic saline (1 ml/100 g bw), the Oxycyte®-group and the HBO + Oxycyte®-group received an i.v. injection of Oxycyte® (1 ml/100 g bw). After injection, breathing was changed to pure oxygen to induce normobaric hyperoxygenation (NBO) in Oxycyte®- HBO- and HBO + Oxycyte®-groups. The NBO treatment was maintained until end of the experiment by keeping the animals in an oxygen tent. In HBO- and HBO + Oxycyte®-group, hyperbaric oxygenation was induced two hours after onset of focal ischemia by placing the animals in a custom-made HBO-chamber where they stayed for one hour. Afterwards they were replaced into the oxygen tent.

Eight hours after MCA occlusion animals were re-anesthetized and after taking an arterial blood sample for blood gas analysis (Omni 4, AVL, Medizintechnik, Bad Homburg, Germany) they were killed by bleeding from the aorta. Brains were immediately removed, frozen in pre-chilled n-pentane and stored at −80°C. The skull and brain base were carefully inspected and animals with incorrect placement of the filament or occurrence of subarachnoid hemorrhage were excluded from further analysis.

### Volumetric analysis of brain damage

From each brain serial coronal sections (thickness, 20 μm) were prepared at −20°C using a cryomicrotom (HM500, Microm GmbH, Wiesloch, Germany). Sections were taken every 500 μm and transferred to poly-l-lysine coated slides. After air drying a silver nitrate staining was performed as described previously (Vogel et al., 1999). Stained sections were scanned and the area of cortical and subcortical ischemic damage along with the entire areas of the left and right hemisphere outlined using Scion image software (free ware available at http://www.scion-image.software.informer.com). Volumes of both hemispheres and of ischemic cell death were calculated taking into account the distance between the sections. The extent of swelling was calculated by the equation described by Kaplan et al. (1991):


### Data analysis

Data are represented as mean ± SD. Statistical analysis was performed using one-way analysis of variance (ANOVA) procedure and subsequent Fisher’s protected least significant difference test. Values of *p* < 0.05 are considered statistically significant.

## Results

Blood gas analyses performed at the end of the 8 hours observation period revealed a significantly increased p_a_O_2_ in NBO-treated animals (>450 mmHg in all groups). Similarly, the drop of the LDF signal after MCAO was not different between the different treatment groups and ranged around 80%, which is comparable to previous findings (Woitzik and Schilling, 2002; Woitzik et al., 2006). Mean arterial blood pressure remained at constant levels throughout the time course of the measurement in all animals. Especially, no changes were observed during infusion of Oxycyte® or saline. All 20 animals (five per group) survived the 8 hours observation period (Table 1).

**Table 1:** **Blood gas analysis at the end of the eight-hours observation period**

	Control (n = 5)	Oxycyte® (n = 5)	HBO (n = 5)	HBO + Oxycyte® (n = 5)
pH	7.45 ± 0.05	7.47 ± 0.07	7.42 ± 0.03	7.47 ± 0.01
p_a_O_2_	75.3 ± 11.7	593.7 ± 64.9	491.5 ± 12.2	509.7 ± 42.5
BaseExcess	0.5 ± 1.9	1.6 ± 2.3	2.9 ± 2.0	2.3 ± 1.2
Hct	47.4 ± 3.5	42.8 ± 3.2	43.7 ± 0.7	47.3 ± 2.1

**Figure 1:**
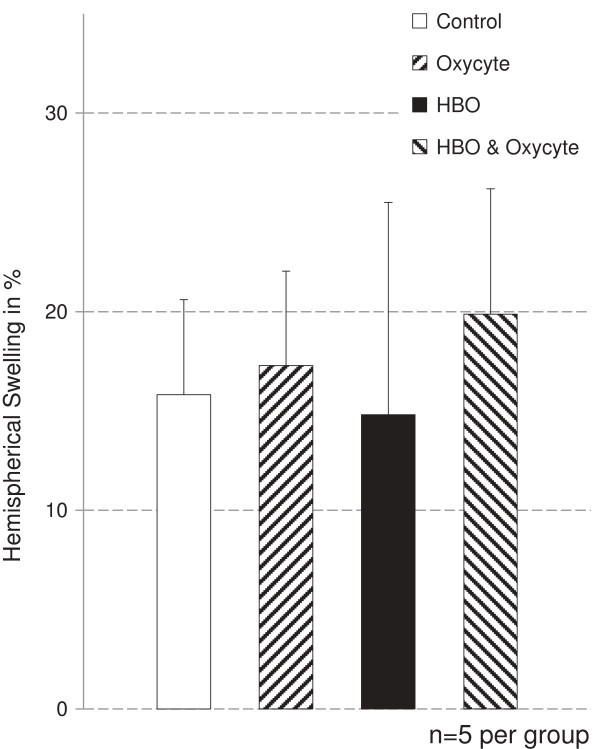
**Hemispherical swelling was calculated in per cent of total volume for correction of necrosis values.** No statistical difference could be found between the groups.

**Figure 2:**
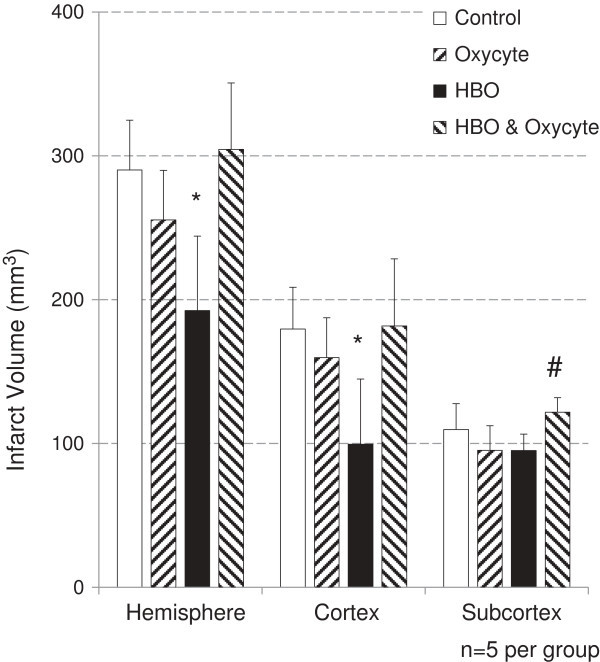
Significantly smaller volumes of infarction are registered in HBO-treated animals in cortical areas, and thus throughout the whole hemisphere (* = p < 0.05 compared to all other groups). Oxycyte®-treated animals show a tendency towards smaller infarction volumes when compared to control-animals. Significantly larger subcortical infarct volumes are registered in HBO + Oxycyte®-treated animals (# = p < 0.05 compared to Oxycyte®- and HBO-group).

The amount of hemispherical swelling did not differ significantly between the treatment groups (Figure 1). Swelling corrected necrotic tissue volumes were not significantly different between Control-animals, Oxycyte®-animals and HBO + Oxycyte®-animals regarding cortical, subcortical or total hemispheric infarct volumes, yet Oxycyte®-treated animals showed a tendency towards smaller infarction volumes when compared to Control-animals (p < 0.1). HBO-animals showed significantly smaller infarct sizes in cortical regions, and thus throughout the whole hemisphere, compared to all other groups. HBO + Oxycyte®-animals had significantly larger infarct sizes in subcortical regions compared to HBO-animals and to Oxycyte®-animals, but not in comparison to Control-animals. (**Hemisphere**: Control 290 ± 35 mm^3^, Oxycyte® 255 ± 35 mm^3^, HBO 192 ± 52 mm^3^, HBO + Oxycyte® 305 ± 46 mm^3^; **Cortical**: Control 180 ± 29 mm^3^, Oxycyte® 160 ± 28 mm^3^, HBO 100 ± 45 mm^3^, HBO + Oxycyte® 182 ± 47 mm^3^; **Subcortical**: Control 110 ± 18 mm^3^, Oxycyte® 95 ± 17 mm^3^, HBO 95 ± 11 mm^3^, HBO + Oxycyte® 122 ± 10 mm^3^) (Figure 2).

## Discussion

The present study failed to show an additional effect of PFC-administration combined with hyperbaric oxygenation with the idea of further improvement of the tissue oxygen supply by enlarging the oxygen-carrying capacity of Oxycyte®.

Sunami and coworkers have induced hyperbaric oxygenation as a therapeutic option early after establishment of focal cerebral ischemia (Sunami et al., 2000). In this study, the oxygen transport capacity of blood was increased by 20%. This increase resulted in an 18% decrease of ischemic brain damage. These values are slightly lower than those found in the present study (33% decrease of tissue necrosis volume in HBO-group compared to control group). The molecular mechanisms that lead to this neuroprotective effect may be various and are still predominantly unknown (Ostrowski et al., 2005; Calvert et al., 2006; Gu et al., 2008; Matchett et al., 2009). The most straightforward theory might be direct neuroprotection via enhancement of oxygen supply to the tissue at risk via collateral vessels (e.g. leptomeningeal arteries).

Another promising therapeutic option is the application of second generation perfluorochemicals (PFCs). Their oxygen dissociation curve is linear depending on the partial oxygen pressure making oxygen transport poor at low p_a_O_2_-values. However, when p_a_O_2_ drops from 550 mmHg (provided by NBO) to 50 mmHg (tissue pressure), PFCs can release 6.9 vol % O_2_. Blood at a haematocrit of 45% can release 5.9 vol % O_2_ over the same pressure drop, which makes them very similar concerning oxygen-carrying capacity (Zauner et al., 2002). The total oxygen-carrying capacity of Oxycyte® is approximately 20 ml × 100 ml^-1^ × bar^-1^. The dose applied, 1 ml per 100 g bw, was considered a moderate dose which added approximately 15% of the total oxygen-carrying capacity to the circulating blood. This volume did not affect arterial blood pressure indicating that it did not exert any significant volume load.

The effects of PFCs on functional parameters such as tissue oxygen availability, recovery of brain electrical activity and reconstitution of neurological function have been studied in different models of transient focal cerebral ischemia (Sutherland et al., 1984; Peerless et al., 1981; Suzuki et al., 1984; Pereira et al., 1988). Also negative results were reported (Kolluri et al., 1986a; Pereira et al., 1988). Yet, an increase of O_2_ availability has been observed in the border zones of ischemic brain areas upon the administration of a PFC solution and increase of inspired oxygen content, indicating that collateral blood flow and PFC flow via the leptomeningeal anastomoses play important roles (Sutherland et al., 1984). The effect of intravenously administered PFCs on the volume of ischemic brain damage following permanent MCAO has previously been shown by our group (Woitzik et al., 2005). Although the total reduction of infarct volume obtained by the administration of Oxycyte® in the present study is very much consistent with the reduction we could show in the past study (10% present study vs. 12% past study), our present results did not meet the level of significance. This may be due to slightly lower volumes of infarction in this study, although the same treatment protocol had been used.

The hypothesis of direct neuroprotection via enhancement of oxygen delivery to the tissue at risk should be tested. The combination of both treatment strategies seemed promising, considering that the amount of physically dissolved oxygen in the plasma is directly related to the partial oxygen pressure in a linear function. With increasing pressure, the physically dissolved oxygen fraction becomes the more and more dominant oxygen source compared to hemoglobin. An additional increase of 1 bar of pressure should have doubled the oxygen-carrying capacity of the applied emulsion to 35–40%. Unfortunately, blood gas analysis devices do only perform accurate measurements within a certain range. A p_a_O_2_ of more than 450 mmHg is well beyond accurate measuring limitations, so that absolute values could not be obtained.

The present results concerning therapeutic HBO and administration of PFCs after permanent MCAO are on the whole consistent with previous findings. Yet, we could not find smaller volumes of infarction in HBO + Oxycyte®-animals. Our hypothesis that combination of both treatment modalities could lead to further decrease of necrotic tissue volume by improving oxygen supply to the tissue at risk could not be approved. Regarding the present data, it is assumable that the neuroprotective mechanism of HBO or PFC is not the direct improvement of oxygen supply, but comprises more complex pathways some of which have already been brought up by other authors and involve the down-regulation of HIF-1α and its target genes BNIP3, a proapoptotic member of the Bcl-2 family and vascular endothelial growth factor (VEGF), which leads to increased permeability of the blood brain barrier (Ostrowski et al., 2005; Calvert et al., 2006; Gu et al., 2008). Furthermore the combination of HBO and Oxycyte® does not only fail to provide an additional benefit, but even seems to neutralize the neuroprotective effect of each of the treatment modalities. We can only speculate on the mechanisms underlying this important finding. Very early during the development of perfluorochemicals, side effects were observed on the mononuclear phagocyte system. This can also be influenced by hyperbaric oxygenation, so that a possible cause for the neutralisation of the benefit of the two treatments might be found in these inflammatory molecular mechanisms (involving heatshock protein 72), which both treatment options have in common (Lutz, 1983; Taylor et al., 2012; Vince et al., 2011). Nevertheless, other systemic effects, which until now might not have been described, can also be the missing link.

## Conclusion

The neuroprotective mechanisms after focal cerebral ischemia are still predominantly unknown. Whereas in many issues the straightforward ideas are often the most promising, in the question of the neuroprotective effects of HBO and PFCs the underlying mechanisms seem to be more complex and intricate and should therefore remain the target of further investigation.
